# Multimodal neuroimaging of locus coeruleus-default mode network connectivity for predicting dexmedetomidine response in chronic insomnia disorder

**DOI:** 10.3389/fpsyt.2026.1718790

**Published:** 2026-02-25

**Authors:** Yongqiong Tao, Yonghong Zhou, Yongtao Tang, Zhouquan Wu, Ying Li, Haifeng Shi

**Affiliations:** 1 Department of Radiology, Jinshan Hospital of Fudan University, Shanghai, China; 2 Department of Radiology, The Third Affiliated Hospital of Nanjing Medical University, Changzhou Second People’s Hospital, Changzhou Medical Center, Nanjing Medical University, Changzhou, China; 3 Department of Anesthesiology, The Third Affiliated Hospital of Nanjing Medical University, Changzhou Second People’s Hospital, Changzhou Medical Center, Nanjing Medical University, Changzhou, China

**Keywords:** chronic insomnia disorder, default mode network (DMN), dexmedetomidine (DEX), locus coeruleus (LC), PCSL

## Abstract

**Objectives:**

To explore whether pre-treatment functional connectivity between the locus coeruleus (LC) and default mode network (DMN), nodal graph-theoretical features, and gray matter volume can identify chronic insomnia patients who respond poorly to dexmedetomidine patient-controlled sleep therapy (PCSL).

**Methods:**

Chronic insomnia patients underwent PCSL with sub-anesthetic dexmedetomidine and were classified as responders or non-responders based on changes in the Pittsburgh Sleep Quality Index. All patients and matched good sleeper received pre-treatment resting-state functional Magnetic Resonance Imaging (MRI) and structural MRI. Functional connectivity, graph metrics, and gray matter volumes of LC and DMN regions were extracted. Predictors were selected using univariate analysis and Least Absolute Shrinkage and Selection Operator regression (LASSO), then used in a multivariate logistic model. Model performance was assessed via Receiver Operating Characteristic (ROC), calibration, decision curve analysis, and bootstrap validation.

**Results:**

No gray matter volume differences were found between responders and non-responders except increased right hippocampal volume in non-responders versus controls. Non-responders showed widespread increased LC-DMN connectivity pre-treatment, especially between right LC and left lateral temporal cortex versus responders. Graph metrics of key nodes (temporoparietal junction, lateral temporal cortex) were significantly reduced in non-responders. Three imaging features were included in the model, achieving an AUC of 0.816 with good calibration and clinical utility.

**Conclusion:**

Pre-treatment LC-DMN functional and network topology differences may underlie variable responses to dexmedetomidine PCSL in chronic insomnia. The multimodal imaging model effectively predicts treatment sensitivity and may guide personalized interventions.

## 1 Introduction

Chronic insomnia disorder (CID) refers to sleep disorder persisting for more than six months, which demonstrates poor response to cognitive behavioral therapy as well as conventional pharmacological agents, such as benzodiazepine and non-benzodiazepine hypnotics. CID severely impairs patients’ quality of life and markedly increases the risk of depression, anxiety, and neurodegenerative diseases (1, 2).

In recent years, the concept of “anesthesia-based insomnia therapy” has gained attention, aiming to improve sleep architecture by inducing a near-natural sleep state. Dexmedetomidine (Dex) is a selective α_2_-adrenergic receptor agonist that produces sedation closely resembling physiological sleep. Based on Dex’s pharmacological profile, Patient-Controlled Sleep with Dex (PCSL) represents an innovative intervention for CID patients. Following preset parameters set by medical personnel for inducing and maintaining sleep, patients use a self-controlled device to administer Dex when they need to initiate sleep (3). Clinical studies showed that PCSL achieves significant efficacy and good tolerability in approximately half of CID patients. However, another half of the CID patients failed to continue treatment due to unsatisfactory outcomes (4, 5). The presence of treatment-unsensitive subtypes suggests unclear neurobiological mechanisms. Identifying these neurobiological mechanisms and pre-treatment biomarkers of treatment responsiveness is essential for CID patient stratification and precision intervention.

Neuroimaging biomarkers provide valuable tools for exploring neurobiological mechanisms and predicting treatment outcomes. Resting-state functional magnetic resonance imaging (rs-fMRI) is a primary modality for studying insomnia and arousal regulation. Functional connectivity analysis reveals synchronous or antagonistic activity patterns between brain regions via blood oxygen level-dependent (BOLD) signals (6). Graph theory-based methods further quantify the topological properties of brain network nodes-such as degree centrality, betweenness centrality, and local efficiency-reflecting their network importance and information processing capacity (7). Combining functional connectivity with graph-theoretical metrics enables identification of aberrant connectivity patterns and assessment of node-level topological changes. Structural measures, such as gray matter volume (GMV), complement these analyses by providing anatomical correlates of functional alterations (8).

The LC is a key brainstem nucleus in sleep-wake regulation. Its widespread noradrenergic projections sustain wakefulness, modulate attention, and regulate sleep, and represent a major target for Dex-induced “near-natural sleep (9). Animal and human studies show that Dex suppresses LC neuronal firing and reduces norepinephrine release, generating electroencephalographic patterns resembling non-rapid eye movement (NREM) sleep (10, 11). The DMN is central to intrinsic cognition, emotional regulation, and sleep quality, which includes medial prefrontal cortex, posterior cingulate cortex, precuneus, and hippocampus. DMN is modulated by the LC via thalamic pathways and is implicated in the “hyperarousal hypothesis” of insomnia (12, 13). Moreover, CID is also linked to altered GMV and degree centrality in multiple DMN hubs, indicating structural and functional deficits that may underlie refractory insomnia persistence (14, 15). Research on LC volumetrics and graph-theoretical metrics in CID is rare, and no study has compared pre-treatment LC-DMN connectivity between Dex responders and non-responders in CID.

Based on these background, the present study focuses on pre-treatment resting-state functional connectivity, graph-theoretical node metrics, and GMV within the LC-DMN pathway in CID patients. A multivariate prediction model was developed to identify potential neuroimaging biomarkers of sensitivity to Dex thereby providing a neurobiological basis for individualized interventions in CID.

## 2 Materials and methods

### 2.1 Study participants

This prospective study was approved by the Ethics Committee of the Third Affiliated Hospital of Nanjing Medical University (Approval No. [2022]YLJSA069). This study is a prespecified substudy within the registered clinical trial (China Clinical Trial Registration No. ChiCTR2300071710). Written informed consent was obtained from all participants. Between June 2022 and June 2023, a total of 72 CID patients were enrolled from the Anesthesia and Sleep Medicine Clinic of the Third Affiliated Hospital of Nanjing Medical University. Inclusion criteria included: diagnosis of CID according to the Diagnostic and Statistical Manual of Mental Disorders, Fifth Edition (DSM-5) and the International Classification of Sleep Disorders, Third Edition (ICSD-3), confirmed by two senior clinicians (W.Z.Q. and Z.Y.H.); age 18–60 years; right-handedness; Han ethnicity; Pittsburgh Sleep Quality Index (PSQI) score >7 (16), total sleep time <7 hours; and sleep onset latency >45 minutes based on actigraphy recording; and insensitivity to conventional pharmacological treatments and cognitive behavioral therapy and completed PCSL treatment. Exclusion criteria included: patients with comorbid depression, indicated by a 17-item Hamilton Rating Scale for Depression (HAMD-17) score ≥7 (17);insomnia disorder secondary to other severe conditions; other types of sleep disorders; patients on CNS-active medications; any other neurological, psychiatric, or somatic disorders; any history of drug or alcohol abuse. Patients discontinued hypnotic medications prior to treatment.

All participants wore a wrist actigraphy device for one week prior to MRI scanning to obtain objective sleep measurements. The devices were continuously worn throughout the treatment period. Actigraphy recorded parameters including time in bed, sleep latency, total sleep time, and sleep efficiency (SE). All participants completed the PSQI and HAMD-17, which were independently scored by two senior neurologists. PSQI assessments were conducted within one week before and within one week after PCSL treatment. The PSQI improvement rate was calculated as follows (18):

ΔPSQI (%) = (PSQI score before treatment−PSQI score after treatment)/ PSQI score before treatment × 100%.

The sample size estimation was based on the anticipated discriminative ability of the prediction model. Considering a multivariable logistic regression model with 80% statistical power (two-sided α = 0.05) to detect an area under the receiver operating characteristic curve (AUC) ≥ 0.68, approximately 30–40 patients per group were required. The final enrolled sample comprised 32 patients in the NR-CID and 40 R-CID, meeting the minimum sample size requirement. With this sample size, the logistic regression model allows inclusion of up to three predictor variables to avoid overfitting (19).

### 2.2 Treatment protocol and follow-up

The Dex-PCSL protocol consisted of two phases: sleep induction and sleep maintenance, strictly following the standardized procedures proposed by Professor Jianxiong An, the founder of the PCSL method (20, 21).

#### 2.2.1 Sleep induction phase

Patients were admitted to a quiet, dimly lit anesthesia sleep room and positioned supine. Intravenous access was established for drug administration. Vital signs, including blood pressure (BP), heart rate (HR), respiratory rate (RR), peripheral oxygen saturation (SpO_2_), and polysomnography (PSG), were continuously monitored to assess sleep stages. Dex (National Drug Approval Number: H20213533; Sichuan Medco Huakang Pharmaceutical Co., Ltd., Chengdu, China; 200 μg) was diluted to 4 μg/mL and infused via a constant-rate syringe pump at 60 mL/h until PSG indicated transition to N2 sleep (sleep spindles and K-complexes). The total dose required to reach N2 sleep was recorded. After Dex titration, patients who experienced discomfort or adverse events, such as transient hypertension, bradycardia, or hypotension, were excluded from the home-based PCSL treatment. Patients who tolerated the procedure without significant adverse reactions proceeded to the PCSL protocol.

#### 2.2.2 PCSL maintenance phase

Before initiation of the home-based PCSL phase, patients received standardized sleep hygiene education and detailed training on the operation of the patient-controlled sleep device. After achieving stability post-induction, patients self-administered Dex under remote monitoring and professional supervision, allowing individualized dose adjustments. The PCSL device (Rehn Medtech Co., Ltd., Jiangsu, China) contained 800 μg Dex diluted in 200 mL saline (final concentration: 4 μg/mL). Device parameters were set as follows: continuous background infusion at 0.1 mL/h (0.4 μg/h), maximum infusion rate 30 mL/h (120 μg/h), single bolus dose 1–3 mL (4-12 μg), with a 10-minute lockout interval following each bolus. Specific parameters were determined based on titration data obtained in the post-anesthesia care unit. The initial dose was individualized according to the amount required for each patient to achieve N2 sleep. Patients could activate the device by pressing a button on a handheld controller, which sent a signal to the microprocessor controlling the syringe pump to deliver the pre-set Dex dose intravenously. Patients were also allowed to self-administer the medication upon nocturnal awakenings. No Dex doses were administered after 4:00 a.m., and any spontaneous awakenings at or after this time were considered part of the natural sleep-wake cycle. Dex doses were individualized and gradually tapered under the supervision of an anesthesiologist. Patients attended regular hospital or community clinic visits for intravenous line replacement and medication preparation.

#### 2.2.3 Follow-up and evaluation of sleep

**Figure 1:**
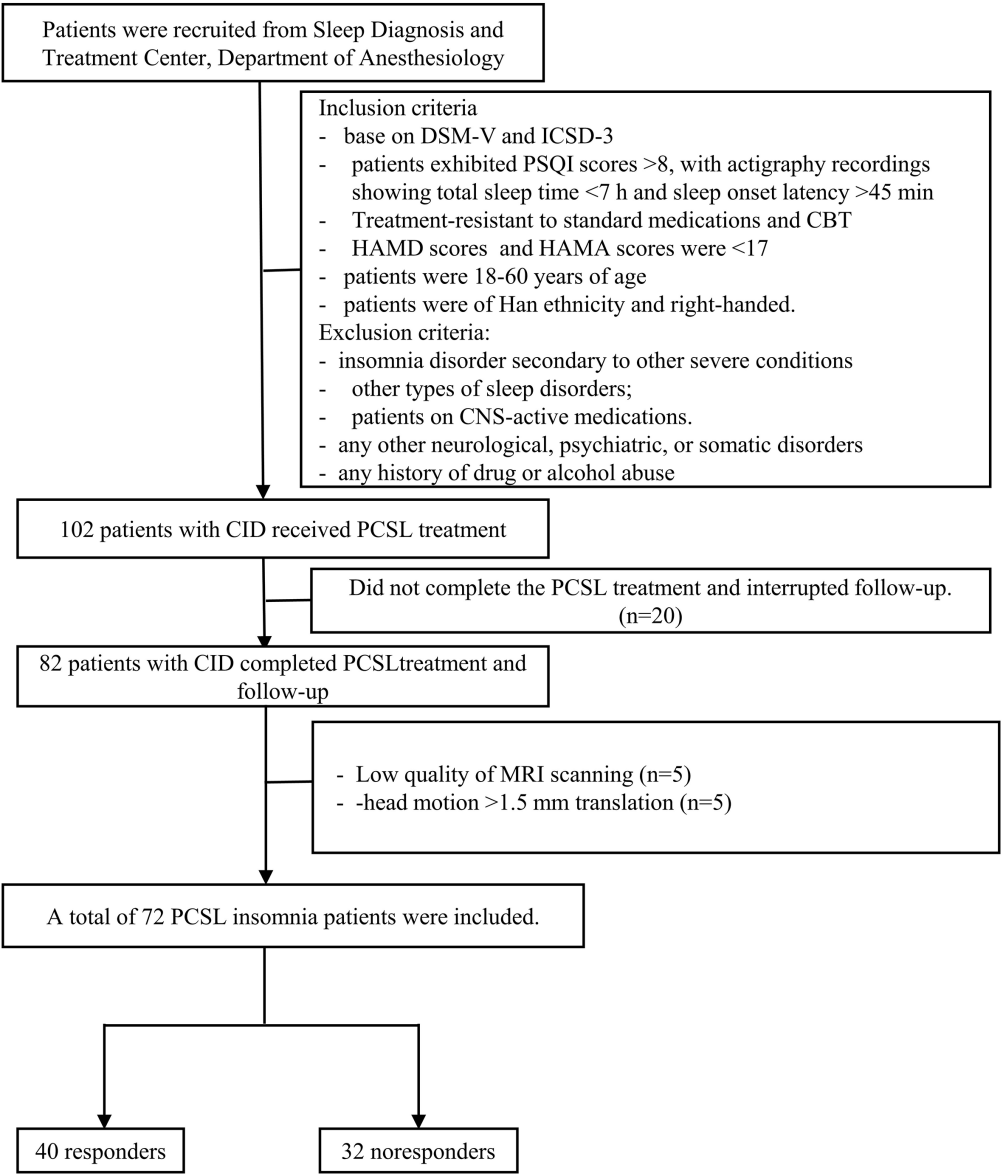
Flowchart. DSM-V, Diagnostic and Statistical Manual of Mental Disorders, version 5; ICSD-3, International Classification of Sleep Disorders, Third Edition; PSQI Pittsburgh Sleep Quality Index; HAMD, Hamilton Depression Scale; CBT, Cognitive Behavioral Therapy; MRI, magnetic resonance imaging; CID Chronic insomnia disorder; PCSL, Patient-Controlled Sleep Therapy using Dexmedetomidine; CNS, Central Nervous System.

The follow-up period lasted 3 months, during which treatment efficacy was evaluated via telephone follow-up and actigraphy. The primary endpoint was improvement in insomnia symptoms, and the secondary endpoint was improvement in depressive symptoms. Based on PSQI improvement after treatment, patients were classified into responders with comorbid insomnia disorder (R-CID, n = 40) and non-responders (NR-CID, n = 32). Additionally, 32 age- and sex-matched healthy volunteers with good sleep quality (GS group) were recruited from the local community as controls. Inclusion criteria for the GS group were PSQI <7, total sleep time >7 hours, and sleep onset latency <30 minutes (as recorded by actigraphy), with exclusion criteria consistent with the CID group. The study flowchart is presented in **Figure 1**.

### 2.3 MRI acquisition

All data were collected using a 3.0 T scanner (Philips Healthcare, Netherlands) with a standard 32-channel head and spine combination coil. During the scan, participants were supine with their eyes closed to minimize external visual stimulation, and their heads were secured with sponge pads to minimize motion artifacts. Rubber earplugs were provided to reduce noise interference. Participants were instructed to stay awake, relax, and avoid active thinking. The main research sequences included rs-fMRI and routine sequences included 3D T1-weighted imaging, T2-weighted imaging, and T2-FLAIR imaging. To rule out the influence of brain atrophy on results, we calculated brain volume (TIV), gray matter volume (GMV), and cerebral white matter lesion volume (WMH) for each participant using 3D T1-weighted imaging. Detailed imaging parameters and methods for calculating brain volumes are provided in the Supplementary Materials.

#### 2.3.1 Image preprocessing and rs-fMRI processing

Data in the DICOM format were first converted using SPM12 (https://www.fil.ion.ucl.ac.uk/spm/software/spm12/), and then preprocessed using DPABI (https://rfmri.org/DPABI). During preprocessing, time correction and head movement correction (Realign) were applied to the EPI images. Data with head motion exceeding 1.5 mm in any direction or rotation exceeding 1.5° were excluded. Additionally, the percentage of time points with mean FD-Power > 0.5 was used as a criterion for motion assessment (22). The FD-Power values for all participants are reported in **Table 1**. The first 10 volumes of the resting-state data were removed to account for signal instability. The remaining data were rearranged, registered, and spatially standardized. A Gaussian kernel with a full width at half maximum (FWHM) of 6 mm was applied to smooth the data. A band-pass filter (0.01-0.08 Hz) was applied to remove low-frequency drift and high-frequency signals such as those caused by respiration and heartbeat. Linear trends were removed, and covariates such as head motion parameters, white matter signals, and cerebrospinal fluid signals were regressed out.

**Table 1:** Demographics, clinical characteristics, and brain volumes of the study participants.

Variable	*R-CIDs (n = 40)*	*NR-CIDs *(n = 32)	GSs (n = 32)	Statistical value	P-value	R-CID vs NR-CID	R-CID vs GS	NR-CID vs GS
Demographics								
Age (year)	47 (20)	39 ± 15.6	47 (27)	*H* = 1.95	0.376	N/A	N/A	N/A
Gender (F/M)	20/20	14/18	17/15	*χ2* = 0.28	0.869	N/A	N/A	N/A
Education (year)	9 (3)	12 (4)	9 (10)	*H* = 0.43	0.805	N/A	N/A	N/A
Duration (year)	3 (9)	3 (9)	N/A	*H =-0.34*	0.732	N/A	N/A	N/A
Scale assessment (baseline)								
HAMD	6(4)	5 ± 1.01	5 (5)	*H* = 1.18	0.554	N/A	N/A	N/A
PSQI	15.33 ± 3.72	13.50 ± 3.71	1 (2)	*H* = 67.86	<0.001***	0.582	<0.001***	<0.001***
Actigraphy data(baseline)								
SOL (min)	60 (30)	60 (90)	10 (10)	*H* = 51.10	<0.001***	1.000	<0.001***	<0.001***
TST (hour)	5 (2)	6 (3)	7 (1)	*H* = 42.25	<0.001***	>0.99	<0.001***	<0.001***
SE (%)	60 (25)	70 (21.5)	95 (10)	*H* = 52	<0.001***	0.025	<0.001***	0.001
TIB (hour)	8 (1)	9 (1)	8 (1)	*H* = 5.82	0.054	N/A	N/A	N/A
Brain volumes								
TIV (mm^3^)	1489 ± 157	1461 ± 124	1417. ± 133	*F = 2.02*	0.138	N/A	N/A	N/A
GM (mm^3^)	646 ± 58	642 ± 51	618 ± 67	*F* = 2.16	0.120	N/A	N/A	N/A
WM (mm^3^)	533 ± 62	528 ± 51	510 ± 48	*F* = 1.39	0.254	N/A	N/A	N/A
CSF (mm^3^)	308 ± 80	289± 57	287 ± 66	*F* = 0.78	0.461	N/A	N/A	N/A
WMH (mm^3^)	0.74 (0.97)	0.69 (0.45)	0.73 (0.45)	*H* = 0.43	0.804	N/A	N/A	N/A
FD-Power(mm)	0.07± 0.01	0.06± 0.02	0.05 ± 0.03	*F =0.78*	0.53	N/A	N/A	N/A

R-CID refers to responders to PCSL in chronic insomnia disorder, NR-CID refers to non-responders, and GS refers to healthy controls with good sleep. HAMD, Hamilton Depression Scale; PSQI, Pittsburgh Sleep Quality Index; SOL, sleep onset latency; TST, total sleep time; SE, sleep efficiency; TIB, time in bed; TIV, total intracranial volume; GM, gray matter; WM, white matter; CSF, cerebrospinal fluid; WMH, white matter hyperintensity; N/A, not applicable; H, Kruskal-Wallis H test; χ2, Chi-square test; F, One-way ANOVA; FD-Power, Framewise Displacement Power 。*P < 0.05 indicates a statistically significant after correction for multiple comparisons. *P < 0.05, **P < 0.01, ***P < 0.001.

#### 2.3.2 ROI definition

**Figure 2:**
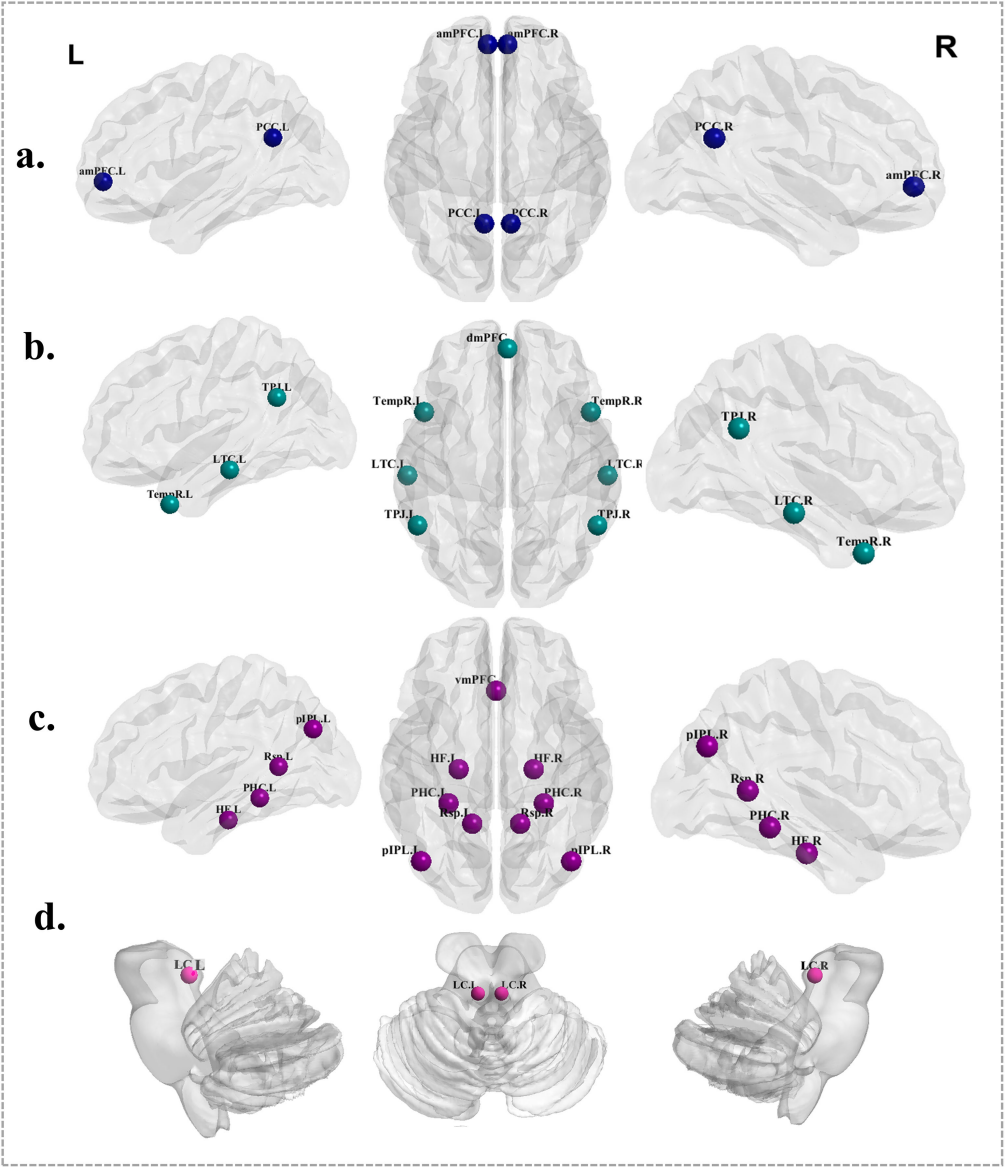
Spatial distribution of regions of interest (ROIs) included in the LC–DMN network. **(a)** Core DMN subsystem, including the anterior medial prefrontal cortex (aMPFC) and posterior cingulate cortex (PCC); **(b)** dMPFC subsystem, including the dorsal medial prefrontal cortex (dMPFC), temporoparietal junction (TPJ), lateral temporal cortex (LTC), and temporal pole (TempP); **(c)** medial temporal lobe (MTL) subsystem, including the ventral medial prefrontal cortex (vMPFC), posterior inferior parietal lobule (pIPL), retrosplenial cortex (Rsp), parahippocampal cortex (PHC), and hippocampal formation (HF); **(d)** bilateral locus coeruleus (LC), defined as spherical ROIs in MNI space.

This study focused on the LC-DMN, including the bilateral LC and 20 ROIs within the DMN. Based on previous literature, the DMN was further subdivided into three functional subsystems, and the constituent brain regions of each subsystem are illustrated in **Figures 2a-c** (23). All ROIs were defined as spherical regions. Given the small size and deep anatomical location of the LC, a sphere with a radius of 3 mm was used for the bilateral LC, whereas a radius of 6 mm was applied to the DMN-related cortical regions to better capture their spatial extent and anatomical characteristics. The Montreal Neurological Institute (MNI) coordinates of all ROIs were derived from authoritative prior studies and were carefully cross-validated against commonly used brain atlases, including the Harvard-Oxford and AAL atlases, to ensure accurate spatial localization (24, 25). In particular, the use of a small-radius (3 mm) spherical ROI for the LC provides high spatial specificity for this small subcortical nucleus, and the selected coordinates are consistent with LC locations reported in studies based on the Duvernoy brainstem atlas and related neuroanatomical investigations (**Figure 2d**). The spatial distribution of all 22 ROIs is shown in **Figure 2**, and detailed MNI coordinates are provided in the Supplementary Materials (**Supplementary Table 1**).

#### 2.3.3 Functional connectivity and graph-theoretical node metrics analysis of the LC and DMN

Based on the predefined ROIs, functional connectivity (FC) networks were constructed using GRETNA software. Each ROI served as a network node, from which the mean time series was extracted. Partial correlation coefficients between pairs of node time series were calculated to define network edges, resulting in a 22 × 22 partial correlation matrix for each subject. These matrices were imported into the Network Analysis module of GRETNA for further processing (26), a sparsity range of 0.16-0.40 with a step size of 0.01 was applied. For each threshold level, we computed nodal topological properties, including degree centrality (Dc), betweenness centrality (Bc), local efficiency (Ne), nodal clustering coefficient (Nc), and nodal shortest path length (Nlp). These metrics were then averaged across the sparsity range to obtain stable nodal characteristics for subsequent statistical analysis.

### 2.4 Statistical analysis

Group comparisons of demographic and clinical variables, regional gray matter volumes, and nodal topological features were performed using IBM SPSS Statistics software (version 26.0; IBM Corp., Armonk, NY, USA). For continuous variables that met normality assumptions, one-way analysis of variance (ANOVA) was applied, whereas the Kruskal-Wallis test was used for non-normally distributed variables. Categorical variables were compared using the chi-square test. For group comparisons involving regional gray matter volumes and nodal topological features, *post hoc* multiple comparisons were corrected using the Bonferroni method to control for type I error. Normally distributed continuous variables are presented as mean ± standard deviation (SD); categorical variables are expressed as frequencies; non-normally distributed continuous variables are presented as median and interquartile range (IQR). All tests were two-tailed, and a p-value < 0.05 was considered statistically significant.

A network-based statistics (NBS) approach (http://www.nitrc.org/projects/nbs/) was applied to the Fisher’s z−transformed correlation coefficients to identify differences in FC between the bilateral LC and 20 DMN−related ROIs (22 ROIs in total) across the three groups. NBS combines cluster-level permutation testing with graph-theoretical connected components, allowing effective control of the family-wise error rate (FWE) in large-scale univariate analyses. Multiple comparisons were controlled at the network level using permutation-based FWE correction with 10,000 permutations, ensuring strict control over the false-positive rate across the network (27). The FC matrix was generated by computing pairwise correlation coefficients among all 22 ROIs, encompassing both intra− and inter−network connections involving the LC and DMN subsystems.

### 2.5 Diagnostic model construction

In the *post hoc* analysis, pre-treatment imaging features showing statistically significant differences between the R-CID and NR-CID groups (P < 0.05) were identified. These features were further subjected to least absolute shrinkage and selection operator (LASSO) regression for feature selection and dimensionality reduction, aiming to minimize multicollinearity and avoid overfitting (28). The selected features were then incorporated into a multivariable binary logistic regression model. Model performance was comprehensively evaluated using the receiver operating characteristic (ROC) curve, calibration curve, and decision curve analysis (DCA), to assess discrimination, calibration, and clinical utility, respectively. Internal validation was performed using 1,000 bootstrap resamples to assess the model’s robustness and generalizability.

## 3 Results

### 3.1 Demographics, brain volumes, and clinical characteristics

The baseline characteristics before treatment, including demographic data, brain volumes, and clinical features of the enrolled patients, are summarized in **Table 1**. No significant differences in demographic data and global brain volumes, were observed among the three groups (all P > 0.05). Compared with GS group, both patient groups (R−CID and NR−CID) exhibited higher PSQI scores and longer SOL, but lower TST and SE (P < 0.001). No significant differences were found among the three groups in HAMD scores or TIB (P > 0.05). At baseline, there were no significant differences between the R−CID and NR−CID groups in most clinical characteristics, except that the R−CID group showed significantly lower SE than the NR−CID group (P = 0.025). Since baseline SE differed significantly between R-CID and NR-CID groups, SE was included as a covariate in analyses of gray matter volume, nodal topological features, and resting-state functional connectivity.

The follow-up clinical characteristics of the patient group one year after PCSL are summarized in **Table 2**. After treatment, the PSQI score and PSQI reduction rate in the R−CID group were significantly lower than those in the NR−CID group (P < 0.001), while TST and SE were significantly higher (P<0.05). No significant differences were found between the two groups in TIB or SOL (P>0.05).

**Table 2:** Follow-up clinical characteristics and pre-post sleep comparisons after PCSL in responders and non-responders.

Variable	*R-CIDs*	*NR-CIDs*	Statistical value (between group)	R-CID vs NR-CID	R-CID (pre-treatment vs post- treatment)	NR-CID(pre-treatment vs post- treatment)
PQSI	7 (6)	11 (5)	*Z=-5.579*	<0.001***	p<0.001***	p=0.069
ΔPQSI (%)	50 (30)	0 (27)	*Z=-7.260*	<0.001***	N/A	N/A
SOL (min)	25 (30)	40 (25)	*Z* = -1.935	0.53	p<0.001***	p=0.001**
TST (hour)	6.5 (5)	5.1 (2)	*Z* = -2.936	0.003**	p<0.001***	p=0.961
SE (%)	89 (13)	65 (28)	*Z* = -5.131	<0.001***	p<0.001***	p=0.784
TIB (hour)	8 (1.5)	9.5 (3)	*Z* = -1.367	0.172	p=0.215	p=0.682

R-CID refers to responders to PCSL in chronic insomnia disorder, NR-CID refers to non-responder. PSQI, Pittsburgh Sleep Quality Index; SOL, sleep onset latency; TST, total sleep time; SE, sleep efficiency; TIB, time in bed. N/A, not applicable. *P < 0.05 indicates a statistically significant after correction for multiple comparisons.*P < 0.05, **P < 0.01, ***P < 0.001.

Compared with baseline, the R−CID group showed significant reductions in PSQI and SOL, and significant increases in TST and SE after treatment (P < 0.001). In contrast, the NR−CID group exhibited only a reduction in SOL (P < 0.001), with no significant changes in other sleep indicators (PSQI, TST, SE) compared to baseline (P>0.05).

### 3.2 Group differences in gray matter volume within the LC and DMN regions

**Figure 3:**
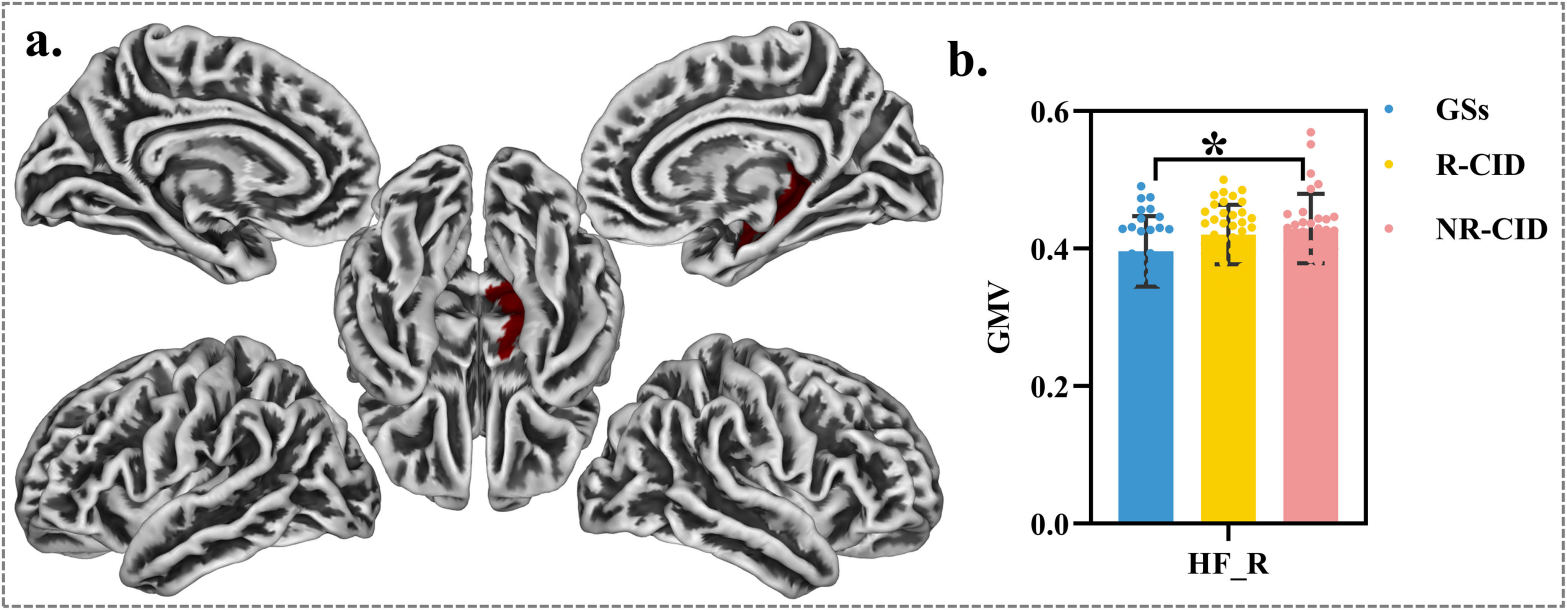
Gray matter volume differences among responders with CID (R-CID), non-responders (NR-CID), and good sleepers (GS). **(a)** Brain map of 22 ROIs; only the right hippocampal formation showed a significant group difference. **(b)** Bar plot of right hippocampal volume (mean ± SE); one-way ANOVA with Bonferroni post hoc (*P < 0.05). Other ROIs were not significant. Abbreviations: GM, gray matter; HF, hippocampal formation; R, right.

The gray matter volumes of 22 brain regions within the bilateral LC and DMN were compared among the groups. No significant differences were found in GMV across the 22 regions between R−CID and NR−CID group, or between R−CID and GS group. However, a significant increase in GMV of the right hippocampal formation (HF_R) was observed in NR−CID compared to GS group (**Figure 3**; P < 0.05).

### 3.3 Connectivity patterns between bilateral LC and DMN in the resting state

**Figure 4:**
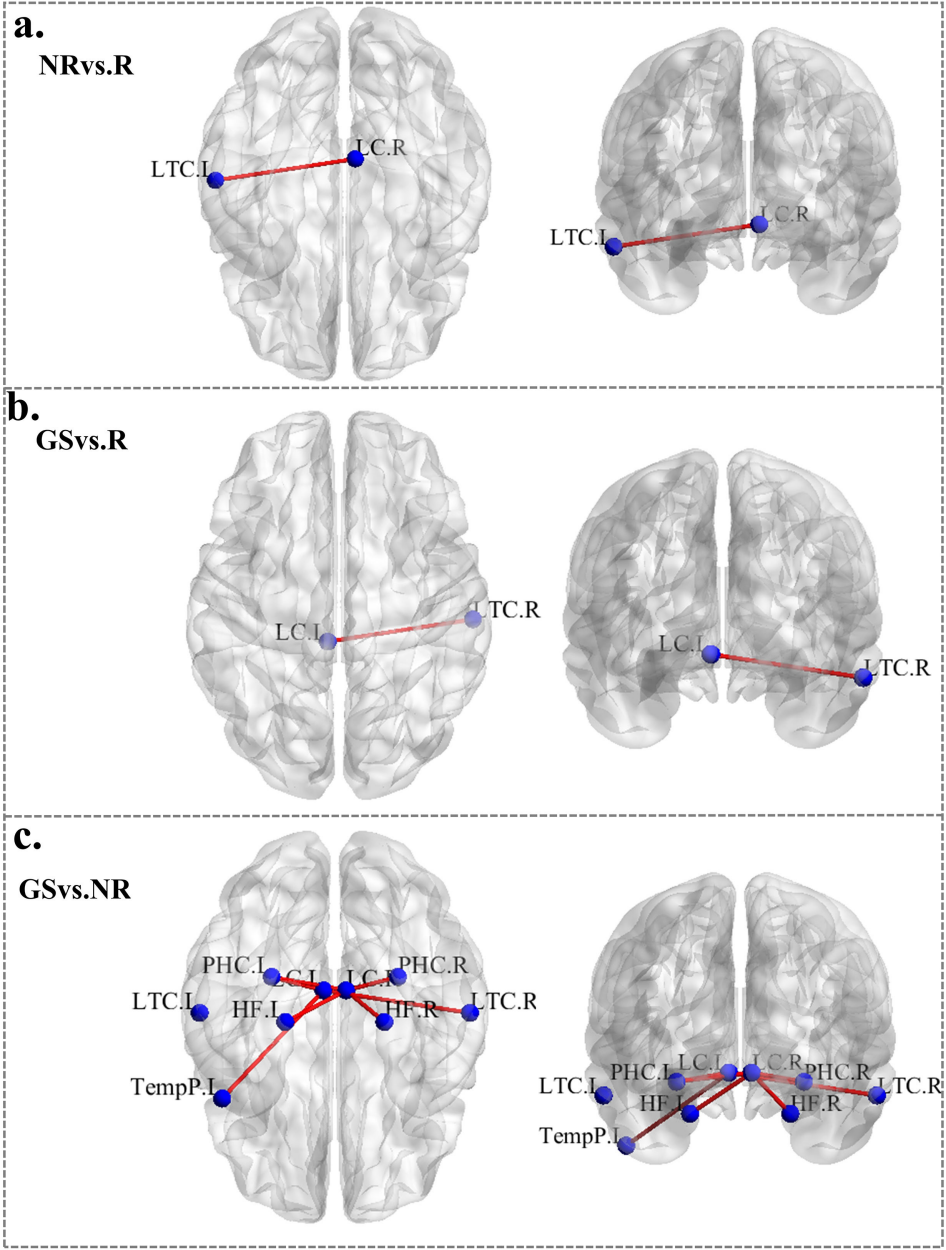
Resting-state functional connectivity differences between the default mode network (DMN) and bilateral locus coeruleus (LC). **(a)** Significantly increased nodal-level static functional connectivity (s-FC) was observed in responders with chronic insomnia disorder (R-CID) compared to non-responders (NR-CID). **(b)** Significantly increased s-FC was found in R-CID compared to good sleepers (GS). **(c)** NS-CID also showed increased s-FC compared to GS. In the connectivity maps, red lines indicate enhanced functional connectivity between specific brain regions. Statistical significance was assessed using Network-Based Statistics (NBS) with P < 0.05. Abbreviations are referenced from **Figure 2**.

Compared with GS group, the NR−CID group exhibited significantly increased functional connectivity in several connections, including LC_L-LTC_R (left LC to right lateral temporal cortex), LC_L-TempR_L (left LC to left temporal pole), LC_L-PHC_R (left LC to right parahippocampal gyrus), LC_R-LTC_L (right LC to left lateral temporal cortex), LC_R-PHC_L (right LC to left parahippocampal gyrus), LC_R-PHC_R, LC_R-HC_L (right LC to left hippocampus), and LC_R-HC_R. (P < 0.05; **Figure 4c**).

Compared with the GS group, the functional connectivity between the left LC and the right LTC was significantly increased in the R−CID group (P<0.05; **Figure4 b**).

Compared with the R−CID group, the NR−CID group showed significantly increased functional connectivity between the right LC and the left LTC (P<0.05, **Figure4 a**).

### 3.4 Topological features of 22 nodes in LC and DMN

**Figure 5:**
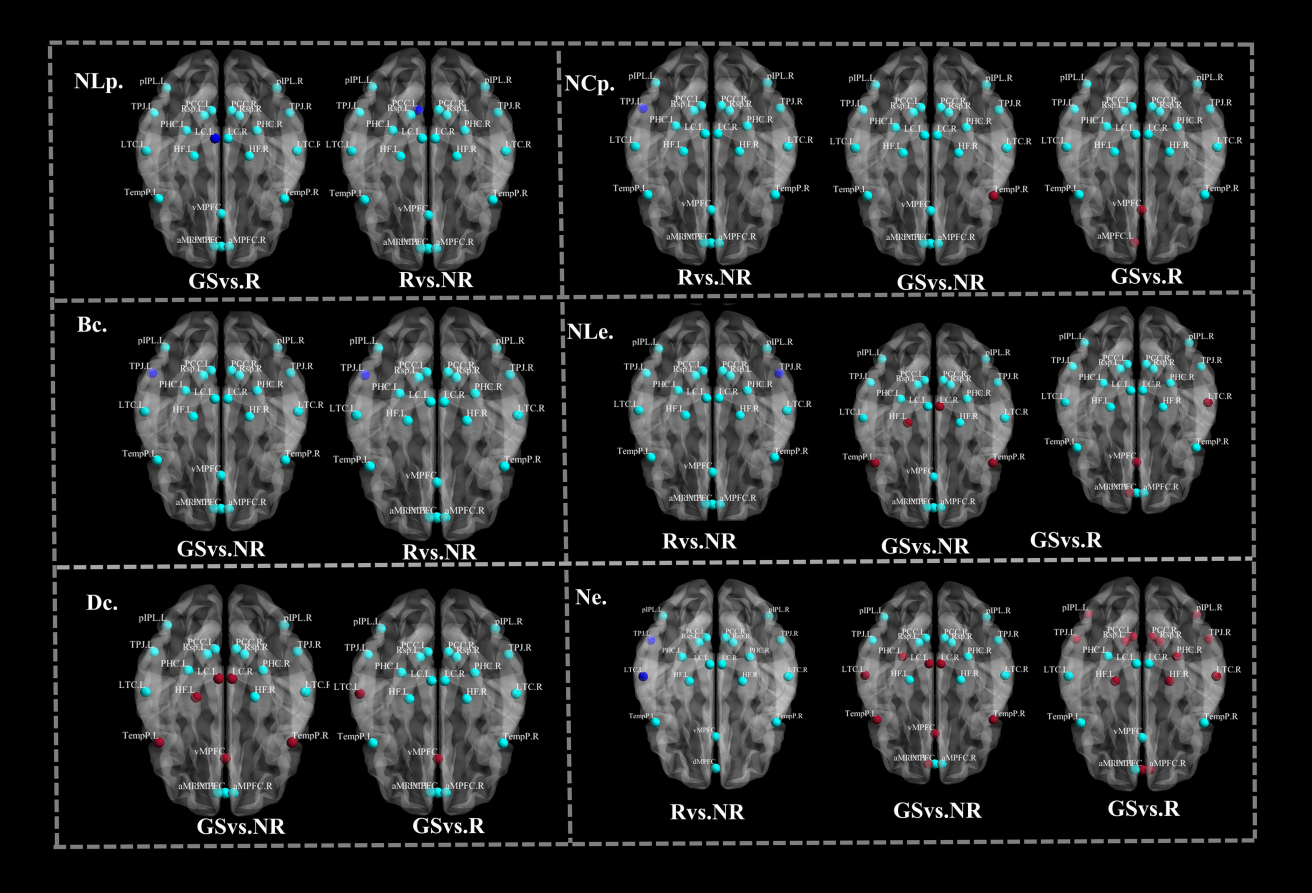
Brain maps showing group differences in nodal characteristics among R-CID, NR-CID, and GS. In the maps, dark blue regions indicate significantly decreased nodal characteristics, red regions indicate significantly increased nodal characteristics, and light blue regions indicate nodes with no significant differences between groups. Nodal characteristics include betweenness centrality (Bc), degree centrality (Dc), nodal clustering coefficient (Ncp), nodal local efficiency (Nlp, Nle), and nodal efficiency (Ne). Statistical significance was determined using Bonferroni post hoc tests (*P <0.05). Abbreviations: Brain region abbreviations are the same as in **Figure 2**.

These results are illustrated in **Figure 5**. Compared with the R−CID group, the NR−CID group exhibited significantly reduced nodal features, including network centrality (Ncp) and local efficiency (NLe) of the right temporoparietal junction (TPJ_R), network efficiency (Ne) and betweenness centrality (Bc) of the left temporoparietal junction (TPJ_L), and network efficiency of the right lateral temporal cortex. (P<0.05).

Compared to GS group, the R−CID group showed significantly increased degree centrality (Dc) in the left lateral temporal cortex (LTC_L) and ventromedial prefrontal cortex (vmPFC), and increased network centrality (Ncp) in the left anteromedial prefrontal cortex (amPFC_L) and vmPFC. Additionally, increased network efficiency (Ne) was observed in the left LC, right LC, amPFC_L, right anteromedial prefrontal cortex (amPFC_R), left posterior cingulate cortex (PCC_L), dorsomedial prefrontal cortex (dmPFC), right temporoparietal junction (TPJ_R), LTC_L, left temporal pole (TempP_L), right temporal pole (TempP_R), vmPFC, left parahippocampal gyrus (PHC_L), and right PHC (PHC_R); while Ne was decreased in the right LTC. Local efficiency (NLe) was significantly increased in the amPFC_L, LTC_R, and vmPFC. Notably, non-local participation (NLp) of LC_L was significantly reduced (all P < 0.05).

In the NR−CID group compared to GS group, betweenness centrality (Bc) of TPJ_L was significantly reduced. Degree centrality (Dc) was elevated in LC_L, LC_R, left temporal cortex (Temp_L), right temporal cortex (Temp_R), vmPFC, and left hippocampal formation (HF_L). Network centrality (Ncp) was higher in Temp_R. Ne was significantly elevated in LC_L, LC_R, amPFC_L, TempP_L, TempP_R, vmPFC, and PHC_L. Additionally, NLe was significantly increased in LC_R, Temp_L, Temp_R, and HF_L (all P < 0.05).

#### 3.4.1 Feature selection and prediction of PCSL treatment sensitivity using LASSO

**Figure 6:**
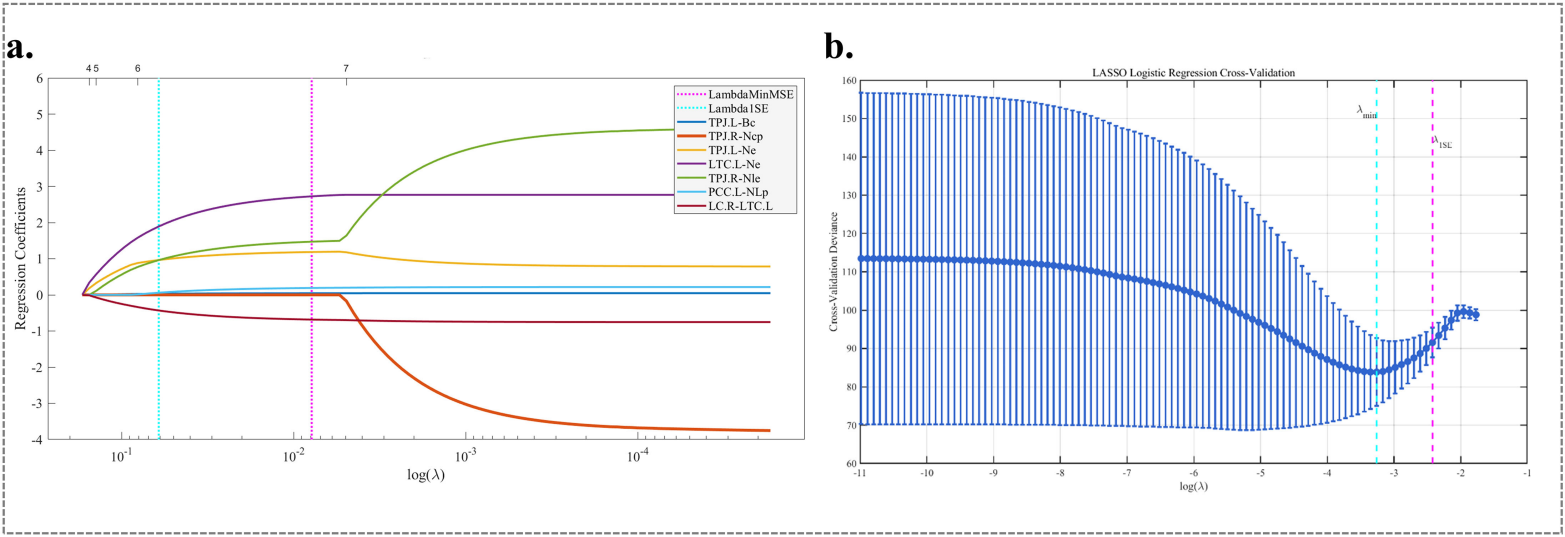
LASSO coefficient paths **(a)** and 10‑fold cross‑validation curve **(b)**. **(a)** Displays imaging feature coefficient trajectories with varying regularization parameter λ. **(b)** Plots 10‑fold cross-validation MSE for each λ (error bars = standard error); vertical dashed lines mark λmin (minimum MSE) and λ1SE (most parsimonious model). Features are selected via LASSO regularization.

Seven variables showing statistically significant differences between R−CID and NR−CID in univariate and *post hoc* analyses were initially included in the LASSO regression (**Figure 6a**). As the regularization parameter Log (λ) increased, the regression coefficients of individual variables gradually shrank toward zero. A 10-fold cross-validation curve was generated (**Figure 6b**), with two vertical dashed lines indicating λ_min (λ = 0.011, Logλ = -4.424), the value corresponding to minimum cross-validation error, and λ_1se (λ = 0.063, Logλ = -2.749), the largest λ within one standard error of the minimum. λ_1se was selected as the optimal penalty parameter to achieve a simpler model with better generalizability. During this process, TPJ_R-Ncp was excluded as its regression coefficient converged to zero, indicating limited independent predictive value. Considering the limited sample size and to reduce the risk of overfitting, only the three most predictive imaging features-TPJ_L-Bc, PCC_L-NLp, and LC_R-LTC_L-were subsequently entered into a multivariate logistic regression model. Based on the results of the multivariate logistic regression analysis, TPJ_L_Bc, PCC_L-NLp, and LC_R-LTC_L were identified as the key imaging features influencing the response to PCSL. Consequently, the formula for predicting the probability of poor response to PCSL (logit [P]) was developed as follows:

Logit(P) = −1.027+0.568 Bc (TPJ_L) + 2.071 NLp (PCC_L) −4.606 FC(LC_R-LTC_L).

Where P represents the probability of poor response to PCSL treatment.

#### 3.4.2 PCSL efficacy prediction model evaluation and validation

**Figure 7:**
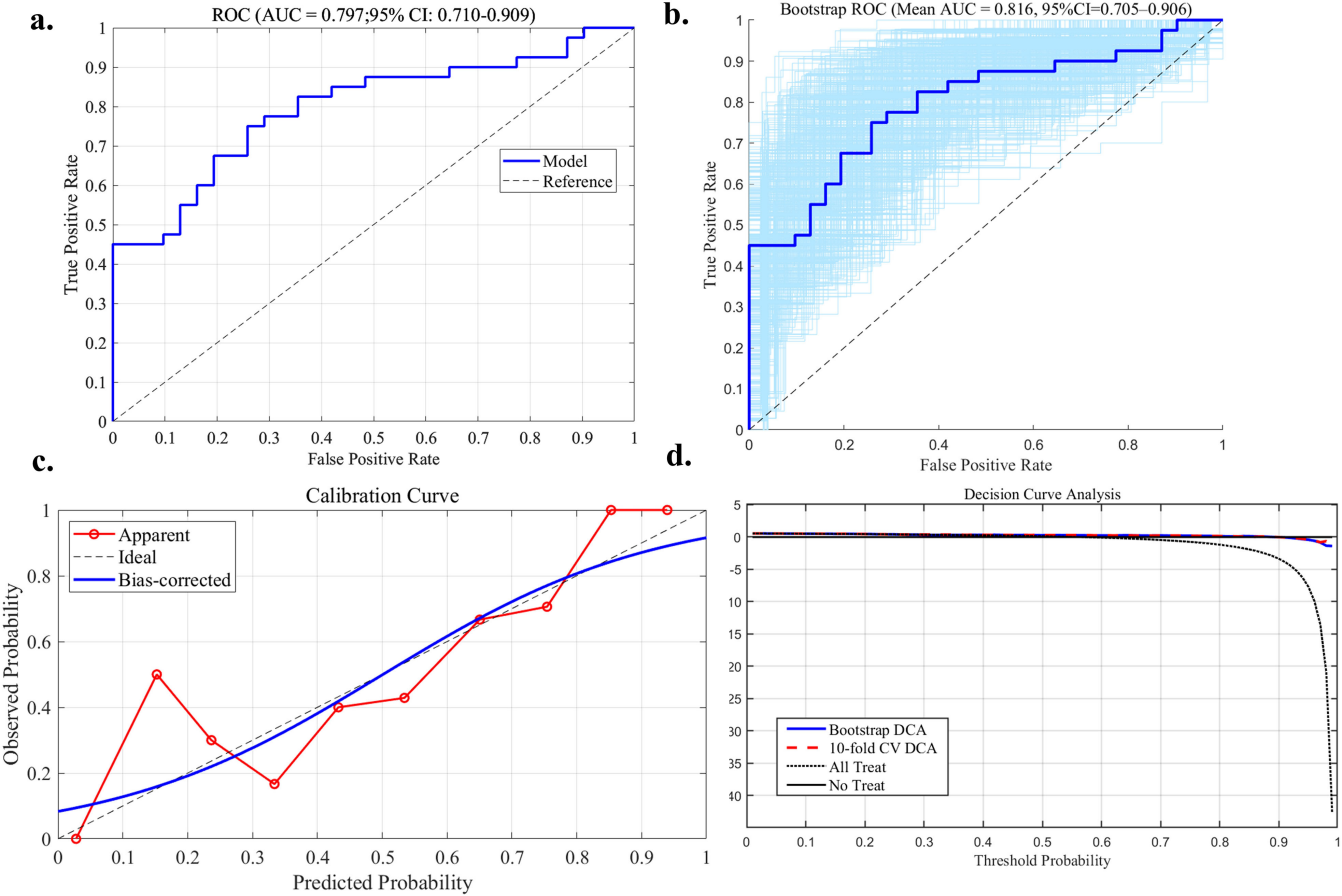
Performance evaluation of the nomogram-based model. **(a)** Receiver operating characteristic (ROC) curve of the model. **(b)** ROC curves derived from 1,000 bootstrap resamples, showing the variability of model performance. **(c)** Calibration curve of the model, comparing predicted and observed probabilities, with the diagonal line representing perfect calibration. **(d)** Decision curve analysis (DCA) of the model, evaluated using 1,000 bootstrap resampling and 10-fold cross-validation (CV), showing the net clinical benefit across a range of threshold probabilities.

The discriminative ability of the nomogram prediction model was evaluated using receiver operating characteristic (ROC) analysis, yielding an AUC of 0.79 (95% CI: 0.71-0.90). After 1,000 bootstrap resamples, the AUC increased to 0.81 (95% CI: 0.70-0.90), indicating good discrimination (**Figures 7a, b**).

To assess calibration, Platt scaling was applied to recalibrate the predicted probabilities. After calibration, Spiegelhalter’s Z-test yielded Z = 0.000 and p = 1.000, suggesting no significant deviation between predicted and observed outcomes. The Brier score of the calibrated model was 0.181, indicating moderate accuracy in probability estimation (**Figure 7c**).

The clinical utility of the model was assessed using decision curve analysis (DCA). Results from 1,000 bootstrap resamples and 10-fold cross-validation showed that the model achieved a high net benefit for predicting response to PCSL treatment within a threshold probability range of 0.05 to 0.95 (**Figure 7d**).

## 4 Discussion

This study investigated individual differences in PCSL response among CID patients and whether pre-treatment LC-DMN structural and functional characteristics relate to outcomes. Using multimodal MRI, gray matter volume, resting-state functional connectivity, and graph-theoretical metrics were assessed, and a predictive model was constructed. Results revealed differences in functional connectivity and network topology, with both patient subgroups showing altered core DMN regions (PCC, TPJ, LTC) compared to good sleepers, suggesting that intrinsic LC-DMN network features may underlie variable treatment sensitivity.

Structural characteristics of specific brain regions—particularly key areas involved in emotional regulation and arousal, such as the hippocampus, prefrontal cortex, and brainstem—may serve as “state markers” or “vulnerability traits” for predicting treatment response (29, 30). Compared with GSs, the NR-CID group showed increased GMV in the right hippocampus, whereas no such difference was observed in the R-CID group. This finding contrasts with previous CID studies reporting hippocampal volume reduction, which may be related to chronic HPA axis activation and neurotoxic effects (31). The increased right hippocampal volume may reflect stress-related neuroplastic or compensatory changes in this subgroup and could serve as a potential biomarker of poor responsiveness to PCSL (32). This structural feature may underlie the attenuated response to Dex-induced naturalistic sleep and warrants further investigation with larger samples to validate its predictive value.

Previous studies have shown that pre-treatment brain structural and functional characteristics can significantly influence therapeutical outcomes. Individual differences in integration capacity, connectivity efficiency, and network topology within key brain regions may reflect sensitivity and adaptability to treatment (33, 34). Subtypes of CID exhibit heterogeneity in DMN connectivity, with hyperarousal-type insomnia often showing increased intra-DMN connectivity, which is associated with difficulties in sleep maintenance and reduced response to conventional treatments (35). In this study, compared with good sleepers, CID patients exhibited enhanced functional connectivity between the LC and cortical regions of the DMN, with NR−CID showing more widespread increases and R−CID showing more limited changes (12, 36). The NR−CID group showed widespread increases in LC-DMN connectivity compared with R−CID, who exhibited more limited connectivity changes. These findings indicate that LC-cortical pathways remain functionally active in insomnia. Physiologically, decreased LC activity reduces norepinephrine projections, disinhibits sleep-active neurons in the preoptic area of the hypothalamus, suppresses ascending arousal systems, and promotes NREM sleep (37, 38). The more extensive LC-DMN coupling in NR−CID may indicate arousal circuit imbalance, highlighting individual differences in sleep regulation and the need for further longitudinal study.

Notably, among all LC-DMN connections, only the pathway between the right LC and the LTC_L showed a significant group difference, with the NR−CID group exhibiting markedly stronger connectivity. The LTC, part of the medial temporal lobe (MTL) subsystem of the DMN, is primarily involved in emotional processing, memory encoding, and context-related self-referential cognition (39). The enhanced LC-LTC functional connectivity in the NR−CID group may represent a hyperarousal-related neural adaptation, reflecting abnormal noradrenergic drive within this pathway. This aberrant activation may impair the sedative mechanism of Dex which relies on suppressing LC neuronal firing and reducing excitatory NE input to the thalamus and cortex to facilitate NREM sleep (40). Clinically, the NR−CID group showed a significant reduction in SOL after intervention, while TST and SE remained unchanged, suggesting that individual differences in brain network characteristics, particularly abnormal LC-LTC connectivity, may limit the efficacy of Dex in maintaining sleep (38). Although this study did not directly assess the relationship between this pathway and clinical outcomes, the findings highlight the need for future functional-behavioral correlation studies.

Graph theory-based brain network analysis allows for the quantitative assessment of the topological properties and potential functional roles of specific brain regions within the whole-brain network. Among node-level metrics, Bc represents the proportion of shortest paths that pass through a given node, reflecting its “hub” role in interregional information transfer. Dc denotes the number of direct connections a node has, indicating its influence within the network. Ne measures the efficiency of a node in integrating information across the brain, while NLe reflects the fault tolerance and information transmission capacity of its local subnetwork. Additionally, Np and NLp further characterize the potential and extent of a node’s information exchange with remote brain regions (41, 42).

Graph-theoretical analysis of key brain regions (bilateral LC and DMN) showed that the NR−CID group exhibited lower network hubness, connectivity, efficiency, and long-range integration compared with the R−CID group. Specifically, the left TPJ, a hub involved in attentional reorienting and self-referential processing, showed significant reductions in Bc, Dc, Ne, NLe, and Ncp, indicating impaired interregional integration and local network stability (43). The left LTC, part of the medial temporal subsystem of the DMN involved in semantic memory and internally directed cognition, showed decreased Ne and Ncp in the NR−CID group, reflecting compromised integration with other DMN subsystems and reduced structural compensation (23). The left PCC, a core DMN hub responsible for large-scale information integration and long-range communication, exhibited significantly lower NLp, suggesting weakened long-range interactions and constrained global network integration (44). Notably, no significant differences were found in any nodal metrics of the bilateral LC, indicating that LC topological features may not directly relate to individual sensitivity to Dex, whereas impaired integration within key DMN regions may limit biomimetic sleep induction. Overall, the NR−CID group showed decreased centrality and integration across multiple arousal-sleep-related regions, potentially impairing transitions from alertness to sleep and providing a neurobiological basis for reduced responsiveness to Dex-induced biomimetic sleep. In contrast, the R−CID group retained more intact topology in LC-DMN-related regions, with higher integration efficiency and nodal centrality, supporting Dex’s activation of LC-cortical pathways and induction of naturalistic sleep (45).

Furthermore, comparisons with GSs revealed that patients with insomnia-regardless of their responsiveness to Dex-exhibited significant alterations in brain network topology within the DMN and LC-related regions. Specifically, the R−CID group showed decreased NLp in the left LC, while Dc, Ncp, and NLe were increased in the LTC_L and vmPFC within the DMN. Additionally, several nodes, including bilateral LC, displayed elevated Ne, indicating integration profiles that diverge from those of healthy individuals. By contrast, although the NR−CID group also exhibited increased Ne or NLe in certain regions (LC, parahippocampal gyrus), key hubs such as TPJ_L showed significantly reduced Bc, and both LC nodes demonstrated elevated Dc and partially increased Ncp-patterns distinct from normal network organization. Importantly, both patient groups demonstrated elevated graph-theoretical metrics across multiple key regions, suggesting widespread brain network remodeling in chronic insomnia irrespective of treatment responsiveness. These alterations may reflect adaptive compensatory mechanisms under prolonged hyperarousal or constitute a neurobiological foundation influencing treatment sensitivity. Therefore, the intrinsic configuration of individual brain networks may serve as a potential predictor of responsiveness to Dex-induced biomimetic sleep (14).

The final nomogram model demonstrated good discriminative ability in differentiating R−CID from NR−CID. After Platt scaling calibration, the Spiegelhalter Z-test indicated excellent agreement between predicted probabilities and observed outcomes. However, considering the limited sample size, the stability of these results requires further validation in larger cohorts. The Brier score was 0.1817, indicating moderate accuracy in probability prediction. DCA further demonstrated favorable clinical utility, showing net benefit across a threshold probability range of 0.05 to 0.95, suggesting the model’s potential applicability in clinical decision-making.

This study has several limitations: the sample size was small and the design was single-center, limiting the generalizability and stability of the predictive model; treatment response was defined solely by PSQI score reduction, lacking objective measures, and future studies should incorporate physiological parameters such as polysomnography; the cross-sectional design precludes evaluation of long-term outcomes; and some potential confounding factors, including prior medication use, psychological status, and comorbid anxiety symptoms, were not fully controlled, which may have affected brain function and treatment response.

Refractory insomnia remains a clinical challenge, with limited efficacy and safety concerns for conventional drugs. Dex offers a novel mechanism for inducing natural-like sleep but shows variable response across patients. Identifying low-responders through neural markers may optimize treatment strategies and resource allocation. This study highlights the LC-DMN pathway as a potential target and provides a preliminary framework for developing individualized prediction tools, supporting precision medicine approaches in sleep disorder management.

## 5 Conclusions

This study found that there are significant inter-individual differences in the therapeutic efficacy of Dex in patients with CID, which may be closely related to differences in functional connectivity patterns between the LC and the DMN, as well as in nodal network topological characteristics. The prediction model constructed based on these multimodal imaging features demonstrated good discrimination, calibration, and clinical applicability, providing both a theoretical basis and a practical tool for the early identification of patients insensitive to PCSL treatment. We are currently collecting new independent sample data to further validate the robustness and external applicability of this model. This process will help assess its reproducibility across different patient populations, ensure reliability and generalizability in clinical practice, and provide a stronger evidence base for optimizing individualized treatment strategies.

## Data Availability

The raw data supporting the conclusions of this article will be made available by the authors, without undue reservation.
