# Correction: Multimodal neuroimaging of locus coeruleus-default mode network connectivity for predicting dexmedetomidine response in chronic insomnia disorder

**DOI:** 10.3389/fpsyt.2026.1818203

**Published:** 2026-03-10

**Authors:** Frontiers Production Office

**Keywords:** chronic insomnia disorder, default mode network (DMN), dexmedetomidine (DEX), locus coeruleus (LC), PCSL

The order of authors in the author list of the published paper was erroneously given as:

“Haifeng Shi1*, Yongqiong Tao1,2, Yonghong Zhou3, Yongtao Tang2, Zhouquan Wu3* and Ying Li1*”

The correct author list reads:

“Yongqiong Tao¹,², Yonghong Zhou³, Yongtao Tang², Zhouquan Wu³*, Ying Li¹*, Haifeng Shi¹*”

The original version of this article has been updated.

