# Supplementary material for: Multimodal neuroimaging of locus coeruleus-default mode network connectivity for predicting dexmedetomidine response in chronic insomnia disorder
Source: Front Psychiatry. 2026 Feb 25;17:1718790. doi: 10.3389/fpsyt.2026.1718790 (PMC13010308; doi:10.3389/fpsyt.2026.1718790)
Supplement: Supplementary file 1 [file Table1.docx]

**The detailed imaging parameters are as follows:**

Routine sequences included T1-weighted imaging, T2-weighted imaging, and T2-FLAIR imaging. Whole-brain blood-oxygen-level-dependent (BOLD) images were acquired using a standard gradient-echo echo-planar imaging sequence with the following settings: repetition time (TR) = 1000 ms, echo time (TE) = 30 ms, acquisition matrix = 68 × 70, matrix size = 3 × 3 mm, flip angle = 90°, field of view (FOV) = 208 × 208 mm, slice thickness = 4.0 mm, number of slices = 36, voxel size = 3 × 3 × 4 mm³, and number of volumes = 240.

**Brain volumes calculation method as follows:**

In this study, the statistical parametric mapping analysis software package 12 (SPM12, version 7771) was run in the MATLAB R2018b environment. The computational anatomy toolbox 12 (CAT12; [http://www.neuro.unijena.de/ cat](http://www.neuro.unijena.de/%20cat)) performed brain volumes analysis on 3D-T1 weighted images. Using the standard protocol, default parameter settings were applied for image preprocessing. Magnetic resonance images in DICOM format were converted to Nii format. Image quality was ensured through visual inspection to check for motion artifacts or other distortions, and corrections were made using the midpoint of the trailing edge to the posterior joint line as the baseline. The longitudinal data preprocessing mode in CAT12 was used to re-align initial images of the same subject at two time points, with intra-subject bias correction performed. Affine normalization based on the East Asian brain template, noise reduction, and intensity inhomogeneity correction were applied. Finally, the images were segmented into gray matter, white matter, and cerebrospinal fluid. Spatial normalization: Segmented images were normalized and registered to the standard space of the Montreal Neurological Institute (MNI) (3) Parameter processing: Brain volume parameters were extracted to obtain total intracranial volume (TIV), gray matter volume (GM), and cerebral white matter lesion volume (WMH).

**Table S1. MNI Coordinates and Radius of the 22 Regions of Interest (ROIs)**

| ROI Name | X (mm) | Y (mm) | Z (mm) | Radius (mm) |
| --- | --- | --- | --- | --- |
| LC.L | -5 | -34 | -8 | 3.0 |
| LC.R | 5 | -34 | -8 | 3.0 |
| aMPFC.L | -6 | 52 | -2 | 6.0 |
| aMPFC.R | 6 | 52 | -2 | 6.0 |
| PCC.L | -8 | -56 | 26 | 6.0 |
| PCC.R | 8 | -56 | 26 | 6.0 |
| dMPFC | 0 | 52 | 26 | 6.0 |
| TPJ.L | -54 | -54 | 28 | 6.0 |
| TPJ.R | 54 | -54 | 28 | 6.0 |
| LTC.L | -60 | -24 | -18 | 6.0 |
| LTC.R | 60 | -24 | -18 | 6.0 |
| TempP.L | -50 | 14 | -40 | 6.0 |
| TempP.R | 50 | 14 | -40 | 6.0 |
| vMPFC | 0 | 26 | -18 | 6.0 |
| pIPL.L | -44 | -74 | 32 | 6.0 |
| pIPL.R | 44 | -74 | 32 | 6.0 |
| Rsp.L | -14 | -52 | 8 | 6.0 |
| Rsp.R | 14 | -52 | 8 | 6.0 |
| PHC.L | -28 | -40 | -12 | 6.0 |
| PHC.R | 28 | -40 | -12 | 6.0 |
| HF.L | -22 | -20 | -26 | 6.0 |
| HF.R | 22 | -20 | -26 | 6.0 |

Abbreviations: LC, locus coeruleus; aMPFC, anterior medial prefrontal cortex; PCC, posterior cingulate cortex; dMPFC, dorsal medial prefrontal cortex; TPJ, temporo‑parietal junction; LTC, lateral temporal cortex; TempP, temporal pole; vMPFC, ventral medial prefrontal cortex; pIPL, posterior inferior parietal lobule; Rsp, retrosplenial cortex; PHC, parahippocampal cortex; HF, hippocampal formation; L, left; R, right.
